# Sociodemographic Trends and Correlation between Parental Hesitancy towards Pediatric COVID-19 Vaccines and Routine Childhood Immunizations in the United States: 2021–2022 National Immunization Survey—Child COVID Module

**DOI:** 10.3390/vaccines12050495

**Published:** 2024-05-03

**Authors:** Olufunto A. Olusanya, Nina B. Masters, Fan Zhang, David E. Sugerman, Rosalind J. Carter, Debora Weiss, James A. Singleton

**Affiliations:** 1 Division of Viral Diseases, National Center for Immunization and Respiratory Diseases, Centers for Disease Control and Prevention (CDC), Atlanta, GA 30333, USA; 2 Center for Biomedical Informatics, Department of Pediatrics, University of Tennessee Health Science Center, Memphis, TN 38103, USA; 3 Epidemic Intelligence Service, Centers for Disease Control and Prevention (CDC), Atlanta, GA 30333, USA; 4 Immunization Services Division, National Center for Immunization and Respiratory Diseases, Centers for Disease Control and Prevention (CDC), Atlanta, GA 30333, USAxzs8@cdc.gov (J.A.S.); 5 Office of the Director, National Center for Immunization and Respiratory Diseases, Centers for Disease Control and Prevention (CDC), Atlanta, GA 30333, USA; 6 Division of Global HIV and TB, Centers for Disease Control and Prevention (CDC), Atlanta, GA 30333, USA

**Keywords:** COVID-19, immunizations, pediatric COVID-19 vaccines, vaccine hesitancy, vaccine confidence, pandemic, National Immunization Survey—Child COVID Module

## Abstract

Multiple factors may influence parental vaccine hesitancy towards pediatric COVID-19 vaccines and routine childhood immunizations (RCIs). Using the United States National Immunization Survey—Child COVID Module data collected from parents/guardians of children aged 5–11 years, this cross-sectional study (1) identified the trends and prevalence estimates of parental hesitancy towards pediatric COVID-19 vaccines and RCIs, (2) examined the relationship between hesitancy towards pediatric COVID-19 vaccines and RCIs, and (3) assessed trends in parental hesitancy towards RCIs by sociodemographic characteristics and behavioral and social drivers of COVID-19 vaccination. From November 2021 to July 2022, 54,329 parents or guardians were interviewed. During this 9-month period, the proportion of parents hesitant about pediatric COVID-19 vaccines increased by 15.8 percentage points (24.8% to 40.6%). Additionally, the proportion of parents who reported RCIs hesitancy increased by 4.7 percentage points from November 2021 to May 2022 but returned to baseline by July 2022. Over nine months, parents’ concerns about pediatric COVID-19 infections declined; however, parents were increasingly worried about pediatric COVID-19 vaccine safety and overall importance. Furthermore, pediatric COVID-19 vaccine hesitancy was more prevalent among parents of children who were White (43.2%) versus Black (29.3%) or Hispanic (26.9%) and those residing in rural (51.3%) compared to urban (28.9%) areas. In contrast, RCIs hesitancy was higher among parents of children who were Black (32.0%) versus Hispanic (24.5%) or White (23.6%). Pediatric COVID-19 vaccine hesitancy was 2–6 times as prevalent among parents who were RCIs hesitant compared to those who were RCIs non-hesitant. This positive correlation between parental hesitancy towards pediatric COVID-19 vaccines and RCIs was observed for all demographic and psychosocial factors for unadjusted and adjusted prevalence ratios. Parent–provider interactions should increase vaccine confidence, shape social norms, and facilitate behavior change to promote pediatric vaccination rates.

## 1. Introduction

Large-scale childhood vaccination programs are among the most effective and cost-beneficial public health interventions [1]. To offer protection against serious childhood illnesses in the United States (U.S.), the Centers for Disease Control and Prevention’s (CDC) Advisory Committee on Immunization Practices (ACIP) recommends the receipt of routine childhood immunizations (RCIs), which are administered based on age [2]. According to the immunization schedule for birth to 6 years, RCIs include vaccines to prevent hepatitis; rotavirus; diphtheria, tetanus, and acellular pertussis (DTaP); *Haemophilus influenzae* type B; pneumococcal conjugate; inactivated polio; influenza; measles, mumps, and rubella (MMR); and varicella [2]. On 29 October 2021, the U.S. Food and Drug Administration (FDA) authorized the Pfizer-BioNTech COVID-19 vaccine for children aged 5–11 years [3], with the first vaccines administered on 2 November 2021. Pediatric COVID-19 vaccines are currently recommended for children aged six months and older [2,4].

Despite the benefits of vaccinations, the COVID-19 pandemic and other factors may have reversed incremental gains in U.S. vaccination rates [5,6,7,8] and caused a sustained decline in global immunization coverage [9,10]. The pandemic has caused large-scale disruptions to the delivery and uptake of immunization services [6,11,12] and negatively impacted vaccine equity among susceptible populations and geographical areas [13,14]. Disruptions in child wellness visits and missed vaccinations may have been due to COVID-19 preventive measures, healthcare disparities, long-term school closures, and shortages in testing modalities and treatment therapies [7]. In 2022, vaccination rates for MMR, DTaP, and varicella vaccines among kindergartners were lower in most states in the U.S. compared to the previous school year (i.e., 2019–2020), while the national MMR vaccination rate fell below the Healthy People 2030 target of 95% for kindergartners [15].

Parents’ reluctance to accept routine childhood vaccines and adhere to immunization schedules also represents a growing challenge for childhood vaccination programs [5,6,7,10]. Vaccine hesitancy is a “delay in the acceptance or refusal of safe vaccines despite availability of vaccination services” and is among the top ten global health threats [16,17]. The spike in vaccine hesitancy during the pandemic [18,19,20] may have been due to the unprecedented speed of development for COVID-19 vaccines, the rapid introduction of these vaccines, and the emergence of new SARS-CoV-2 variants [21,22,23]. Moreover, COVID-19 vaccine misinformation [8], vaccine mandates, digital hyperconnectivity, and ongoing highly politicized debates surrounding COVID-19 vaccines may have contributed to vaccine hesitancy [23]. Parents’ psychosocial factors (e.g., concerns about vaccine safety and effectiveness, mistrust in public health institutions, and belief-based extremism) and access barriers may also be linked to the reluctance to vaccinate children [24].

These complex and dynamic influences (i.e., pandemic disruptions, vaccine hesitancy, the politicization of COVID-19 vaccines, and parental mistrust) could produce unintended consequences that increase parental hesitancy for other childhood vaccines. It is crucial that emerging vaccine concerns within the context of the COVID-19 pandemic are identified and addressed. This cross-sectional study analyzed data from the National Immunization Survey—Child COVID Module (NIS-CCM), which is representative of the U.S. population. Our study had three objectives. We sought to (1) identify the trends and prevalence estimates of parental hesitancy towards pediatric COVID-19 vaccines and RCIs among parents with children aged 5–11 years over 9 months, (2) examine the relationship between parental hesitancy toward pediatric COVID-19 vaccines and hesitancy towards RCIs, and (3) assess the trends in parental hesitancy towards RCIs over the 9-month study period by sociodemographic characteristics and behavioral and social drivers of childhood COVID-19 vaccination.

## 2. Materials and Methods

### 2.1. Survey Methods

This cross-sectional study analyzed data from the NIS-CCM, which offers population-based, state, and local area vaccination estimates, using interviews conducted between November 2021 and July 2022. The NIS-CCM’s purpose, beginning in July 2021, is to provide estimates of COVID-19 vaccination coverage, parental intent to vaccinate their children against COVID-19, and behavioral and social indicators related to COVID-19 vaccination in children aged 6 months to 17 years [25,26,27]. Since November 2021, the NIS-CCM has expanded to include children aged 6 months to 4 years [26]. The NIS-CCM uses a random-digit-dialed sample of cellular numbers belonging to households with children aged 6 months to 17 years. For our study, these analytic data were restricted to households with children aged 5–11 years. The NIS-CCM survey is administered to an adult in a household who is knowledgeable about a child’s vaccination history (hereafter referred to as a parent) [28,29]. For households with more than one child, the NIS-CCM randomly selects a child in that household to be the referent. Quarterly telephone samples from 4–5-week periods are compiled into a dataset for timeliness and analytic purposes. The data are then weighted to be representative of children in the U.S. population for analysis. The NIS-CCM is conducted in compliance with applicable federal laws and CDC policies and is determined by CDC to constitute public health surveillance. For our study, pediatric COVID-19 vaccine hesitancy is defined as parental hesitancy towards pediatric COVID-19 vaccines.

### 2.2. Measures

The NIS-CCM uses the Behavioral and Social Drivers of Vaccination (BeSD) framework to assess socio-behavioral factors influencing pediatric COVID-19 vaccinations [30]. The BeSD proposes four domains that influence the acceptance and uptake of vaccines: (1) what people think and feel about vaccines; (2) social processes and norms that influence vaccinations; (3) environmental context, practical issues, and available resources; and (4) individual motivations (or hesitancy) that shape vaccination behavior. The BeSD domains are presented below with their associated questions.

#### 2.2.1. Outcome Variables

##### Individual Motivations (or Hesitancy)

Our study outcome measures were (1) parental hesitancy toward pediatric COVID-19 vaccines and (2) parental hesitancy towards routine childhood immunizations (RCIs). For this study, responses to these outcome measures were dichotomized to aid meaningful interpretation.

Pediatric COVID-19 vaccine coverage was determined by asking parents if their children had received at least one dose of a COVID-19 vaccine. Among parents whose eligible children had not yet received any vaccine, parental hesitancy towards the COVID-19 vaccine was assessed with the question, “Once your child is eligible, how likely are you to get [child] a COVID-19 vaccine?” Parents’ responses were dichotomized into: “hesitant” (i.e., unvaccinated [child], definitely will not get a vaccine for [child], and probably will not get a vaccine for [child]) and “non-hesitant” (i.e., definitely will get a vaccine for [child] and probably will get a vaccine for [child]). Parents who reported that they were “not sure” about getting the vaccine for [child] or that their child had received “at least one dose of COVID-19 vaccine” were categorized into the “non-hesitant” group. To assess parental hesitancy towards RCIs, parents were asked, “How hesitant about childhood shots would you consider yourself to be?” Parents’ responses were dichotomized into two categories for this analysis: “hesitant” (i.e., somewhat hesitant and very hesitant) and “not hesitant” (i.e., not that hesitant and not at all hesitant).

#### 2.2.2. Predictor Variables

##### What People Think and Feel about Vaccinations

Parents’ psychosocial characteristics, including confidence in vaccine safety and benefits, beliefs, perceptions, and regrets, were assessed. Parents’ perception of risk for pediatric COVID-19 infections was measured with “How concerned are you about [child’s name] getting COVID-19?” Parents’ perception of vaccine importance against pediatric COVID-19 infection was measured by asking, “How important do you think getting a COVID-19 vaccine is to protect [child’s name] against COVID-19?” Perception of vaccine safety was assessed with the question, “How safe do you think a COVID-19 vaccine is for [child’s name]?” Parental regret was measured with, “If I do not get [child’s name] a COVID-19 vaccine, I will regret it”.

##### Social Processes and Norms That Influence Vaccinations

This domain measures constructs such as family and social influences, peer norms, and health worker recommendations. Associated questions include, “If you had to guess about how many of your family and friends have gotten a COVID-19 vaccine for their children ages 5–11 years?”, “Has a doctor, nurse, or another health professional ever recommended that you get a COVID-19 vaccine for [child’s name]?” and “Does [child’s name]’s school require a COVID-19 vaccine to attend in-person classes?”

##### Practical Issues and Available Resources

Practical issues influencing pediatric COVID-19 vaccine uptake, such as knowledge, affordability, ease of access, vaccination availability, etc., were measured with questions such as, “In the past 7 days, how often has [child’s name] worn a mask when going into indoor public spaces like schools, stores, etc.?”

##### Other Sociodemographic Measures

In addition, NIS-CCM collects sociodemographic information such as the child’s age, race/ethnicity, relationship of survey respondent to child, mother’s educational level, family income, and metropolitan statistical area of residence. Other sociodemographic characteristics assessed include family income and poverty level, number of children under 18 residing in a household, Social Vulnerability Index of county of residence, and Health and Human Services region.

### 2.3. Statistical Analyses

Our study analyzed parents’ or guardians’ interview responses from 1 November 2021 through 31 July 2022 [29]. For all analyses, the denominator consisted of all parent survey interviews completed for children aged 5–11 years each month over the course of 9 months. The NIS-CCM accounts for households without cellular phones, as well as variations in sampling, under-representation, and non-response by weighing and adjusting the data [25]. Additionally, survey weights are calibrated to the reported number of children receiving at least one dose of the COVID-19 vaccine by region based on administrative data reported to the CDC by jurisdictions [26].

Descriptive statistics were used to identify trends and estimates for parental hesitancy towards pediatric COVID-19 vaccines and RCIs over the 9-month period. Furthermore, we examined the prevalence and trends of parental perceptions regarding COVID-19 vaccine safety/importance and pediatric COVID-19 infection. We also compared prevalence estimates of demographic and psychosocial characteristics stratified by the study outcome variables (i.e., parental hesitancy towards the pediatric COVID-19 vaccine and parental hesitancy towards RCIs). Nonoverlapping 95% confidence intervals (CI) determined statistically significant estimates between groups.

A logistic regression analysis investigated the relationship between parents’ hesitancy towards pediatric COVID-19 vaccines and their hesitancy towards RCI. SUDAAN version 11.0.3 was used to account for the complex survey design. Estimates were quantified as proportions, prevalence ratios (PR), and 95% CIs. During the logistic regression analysis, the PR was calculated as the prevalence of pediatric COVID-19 vaccine hesitancy within the RCIs hesitant group divided by the prevalence of pediatric COVID-19 vaccine hesitancy within the RCIs non-hesitant group of parents across different levels of demographic and psychosocial characteristics. The adjusted PR controlled for socio-demographic variables (child’s age and race, mother’s educational level, metropolitan statistical area (MSA) status, and poverty status). Results were determined to be statistically significant if the *p*-value was less than 0.05.

We also utilized weighted linear regression models to analyze the temporal trends in parental RCIs hesitancy levels stratified by sociodemographic characteristics and behavioral and social drivers of the child’s COVID-19 vaccination. The models examined the linear trends in the prevalence of RCI hesitancy for each subgroup, with the estimated slopes from the regression models representing the average monthly percentage point change in the prevalence of RCIs hesitancy.

## 3. Results

During the 9-month study period, 54,329 parents or guardians completed the NIS-CCM interviews. The cumulative response rate for the NIS-CCM through July 2022 was 20.4%. The percentage of children who “received at least one dose of the COVID-19 vaccine” increased by 21.6 percentage points (from 11.6% to 33.2%) during this period.

### 3.1. Prevalence Trend of Parental Hesitancy for COVID-19 Vaccines and RCIs over 9 Months

**Figure 1:**
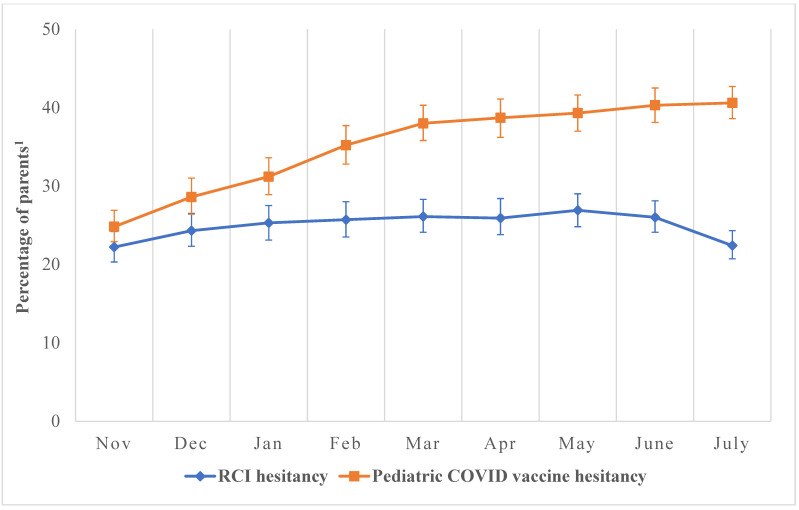
Prevalence of hesitancy towards pediatric COVID-19 vaccines and routine childhood immunizations as reported by parents of children aged 5–11 years, November 2021–July 2022, National Immunization Survey. Abbreviations: RCIs: Recommended Childhood Immunizations. The denominator of the percentages on the *y*-axis represents all completed parent interviews for children aged 5–11-years collated monthly between November 2021 and July 2022. (a) *Pediatric COVID-19 vaccine hesitancy* was assessed with, “Once your child is eligible, how likely are you to get a COVID-19 vaccine?” Responses were “definitely not getting vaccine”, “probably not getting vaccine”, “probably getting vaccine”, and “definitely getting vaccine”. Responses were dichotomized into two categories: *hesitant* (i.e., unvaccinated [child], definitely not getting vaccine for [child], and probably not getting vaccine for [child]) and *non-hesitant* (i.e., definitely getting vaccine for [child] and probably getting vaccine for [child]). The numerator for *pediatric COVID-19 vaccine hesitancy* in Figure 1 represents parents who reported their children were unvaccinated, probably not getting the vaccine, or definitely not getting the COVID-19 vaccine. (b) *RCIs hesitancy* was assessed with, “How hesitant about childhood shots would you consider yourself to be?” Responses were “not at all hesitant”, “not that hesitant”, “somewhat hesitant”, and “very hesitant”. Responses were dichotomized into two categories: *hesitant* (i.e., somewhat hesitant and very hesitant) *and non-hesitant* (i.e., not that hesitant and not at all hesitant). The numerator for *RCIs hesitancy* in Figure 1 represents parents who have reported they were somewhat hesitant or very hesitant towards getting the RCIs for their children. ^1^ The data obtained from the NIS-CCM are weighted to be representative of children in the US. Accordingly, our results reflect the “percentage of children with a parent who has reported hesitancy either towards pediatric COVID-19 vaccines or RCI”. However, to make interpretation easier, we have simplified this as “percentage of parents” on the *y*-axis.

Parents reported hesitancy towards pediatric COVID-19 vaccines and RCIs for children aged 5–11-years, as shown in Figure 1, depicting the trend over 9 months. The percentage of parents or guardians who expressed pediatric COVID-19 vaccine hesitancy and indicated they would “definitely not get” or “probably not get” their children vaccinated against COVID-19 steadily increased by 15.8 percentage points (24.8% to 40.6%) over the 9 months (Figure 1). Over the initial 7-month period, the percentage of parents or guardians who expressed RCIs hesitancy by stating they were “very hesitant” or “somewhat hesitant” towards administering RCIs to their children rose by 4.7 percentage points (22.2% in November 2021 to 26.9% in May 2022). However, this upward trend declined by 3.6 percentage points (26.0% to 22.4%) from June to July 2022 (Figure 1).

**Figure 2:**
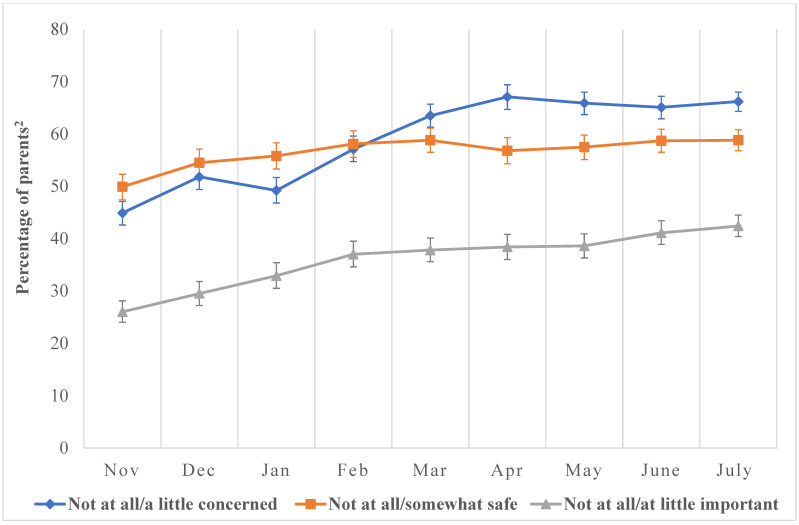
Prevalence of perceptions towards pediatric COVID-19 vaccines as reported by parents of children ages 5–11 years, November 2021–July 2022, National Immunization Survey. The denominator of the percentages on the *y*-axis represents all completed parent interviews for children aged 5–11-years collated monthly between November 2021 and July 2022. The numerator represents parents reporting the level of sentiments and perceptions indicated in the graph (e.g., those whose parents reported that COVID-19 vaccines were not at all or somewhat safe, not at all or a little important, etc.). (a) *Not at all concerned/a little*: We assessed parents’ *concern for pediatric COVID-19 infections* with, “How concerned are you about your child getting COVID-19?” Responses were not at all concerned, a little concerned, moderately concerned, and very concerned. Responses were dichotomized into two categories: Not at all concerned and a little concerned. (b) *Not at all/somewhat safe*: We assessed parents’ *perceptions about COVID-19 vaccine safety* with, “How safe do you think COVID-19 vaccine is for child?” Responses were not at all safe, somewhat safe, very safe, and completely safe. Responses were dichotomized into two categories: Not at all safe and somewhat safe. (c) *Not at all important/a little important*: We assessed parents’ *perception of COVID-19 vaccine importance* by asking, “How important do you think getting a COVID-19 vaccine is to protect child against COVID-19?” Responses were not at all important, a little important, somewhat important, and very important. Responses were dichotomized into two categories: Not at all important and a little important. ^2^ The data obtained from the NIS-CCM are weighted to be representative of children in the US. Accordingly, our results reflect the “percentage of children with a parent who has reported hesitancy either towards pediatric COVID-19 vaccines or RCI”. However, to make interpretation easier, we have simplified this as “percentage of parents” on the *y*-axis.

Throughout the study, parents of children aged 5–11-years expressed fewer concerns about pediatric COVID-19 infections but showed increasing worry about the safety and overall importance of pediatric COVID-19 vaccines (Figure 2).

### 3.2. Prevalence Estimates of Pediatric COVID-19 Vaccine Hesitancy among Socio-Demographic Groups over 9 Months

Pediatric COVID-19 vaccine hesitancy was more prevalent among parents or guardians of children who were White (43.2%) than among Black (29.3%), Hispanic (26.9%), Asian (11.7%), and other/multiple races (35.5%). In addition, mothers with a high school education or equivalent (40.6%) were more hesitant about pediatric COVID-19 vaccines than those with a college degree (28.4%), and parents residing in rural areas (51.3%) were more hesitant than those in urban (28.9%) areas (Table 1).

Parents who were “not at all concerned” or a “little concerned” (48.0%) about pediatric COVID-19 infections were more hesitant towards pediatric COVID-19 vaccines compared to those who were “moderately concerned” or “very concerned” (16.8%). Similarly, the prevalence of COVID-19 vaccine hesitancy was higher among parents who believed that pediatric COVID-19 vaccines were “not at all safe” or “somewhat safe” (55.5%) compared to “very safe” or “completely safe” (5.0%) and among parents who perceived that COVID-19 vaccines were “not at all important” or “a little important” (78.9%) compared with “somewhat important” or “very important” (10.2%). Parents of children aged 5–11 years were more hesitant about pediatric COVID-19 vaccines if they had “none” or “some” family/friends (47.7%) with vaccinated children versus if they had “many” or “almost all” family/friends (7.0%) with vaccinated children. (Table 1).

### 3.3. Prevalence Estimates of RCIs Hesitancy among Socio-Demographic Groups over 9 Months

The prevalence of RCIs hesitancy was higher among parents of children who were Black (32.0%) compared to Hispanic (24.5%), White (23.6%), Asian (18.8%), and other/multiple races (25.8%). Parents residing in rural areas (30.1%) were more hesitant about RCIs than those in urban areas (24.4%). Parents who believed pediatric COVID-19 vaccines were “not at all safe” or “somewhat safe” (36.4%) were more likely to show RCIs hesitancy compared to those who perceived these vaccines as “very safe” or “completely safe” (8.4%). Similarly, those who considered COVID-19 vaccines as “not at all important” or “a little important” (38.8%) were more hesitant about RCIs than those who thought COVID-19 vaccines were “somewhat important” or “very important” (16.9%). Parents with “none/some” family and friends who had vaccinated their children (30.0%) were more hesitant about RCIs than those with “many/almost all” family and friends (12.6%) (Table 1).

### 3.4. The Relationship between COVID-19 Vaccine Hesitancy and RCIs Hesitancy over 9 Months

Overall, pediatric COVID-19 vaccine hesitancy was approximately 2–6 times as prevalent among parents who were hesitant towards RCIs compared to the RCIs non-hesitant group. This positive correlation was observed across all demographic and psychosocial factors for unadjusted and adjusted prevalence ratios (PRs) (Table 2). For instance, among parents of children who were Asian, pediatric COVID-19 vaccine hesitancy was approximately three times more prevalent among those with RCIs hesitancy compared to those without RCIs hesitancy for unadjusted PR (3.30, CI 2.13–5.10) and adjusted PR (2.97, CI 1.99–4.44) analyses.

Notably, parents with *lower* levels of pediatric COVID-19 vaccine hesitancy in both RCIs hesitant and RCIs non-hesitant groups tended to have higher PR (the PR was calculated as the prevalence of pediatric COVID-19 vaccine hesitancy within the RCIs hesitant group divided by the prevalence of pediatric COVID-19 vaccine hesitancy within the RCIs non-hesitant group of parents) estimates (Table 2). For instance, among parents who expressed regret for not having their children vaccinated (unadjusted PR 6.56, CI 4.53–9.50), pediatric COVID-19 vaccine hesitancy rates were relatively low at 13.1% among RCIs hesitant groups and 2.0% among RCIs non-hesitant groups. In comparison, parents with *higher* pediatric COVID-19 vaccine hesitancy levels in both RCIs hesitant and RCIs non-hesitant groups had lower PR estimates. For example, among parents of children residing in rural areas (unadjusted PR 1.57, CI 1.46–1.70), pediatric COVID-19 vaccine hesitancy rates were relatively high at 68.3% and 43.5% for RCIs hesitant and RCIs non-hesitant groups (Table 2). These findings were consistent for both unadjusted and adjusted analyses. Other socio-demographic indicators that indicated low levels of PR estimates but high levels of pediatric COVID-19 vaccine hesitancy included parents of children aged 5–11 years living below the poverty level (adjusted PR 1.48, CI 1.30–1.69) and those who believed that COVID-19 vaccines were unsafe (adjusted PR 1.35, CI 1.29–1.40) and unimportant (adjusted PR 1.13, CI 1.10–1.16).

### 3.5. Temporal Trends in RCIs Hesitancy by Sociodemographic Characteristics and Behavioral and Social Drivers

Table 3 represents the results of the weighted linear regression models. Over the 9-month period, no linear trends were apparent for RCIs hesitancy for most of the demographic, behavioral, and social characteristics examined. However, parental hesitancy towards RCIs decreased by 1.2 percentage points per month over the 9-month study period among children who never, rarely, or sometimes wore masks in the past 7 days. Also, parental RCIs hesitancy increased by 0.6 percentage points per month over the 9-month study period among children whose parents had received a recommendation for their child’s COVID-19 vaccination from a healthcare provider.

## 4. Discussion

Our study investigated trends in parental hesitancy towards pediatric COVID-19 vaccines and RCIs over 9 months and examined the relationship between pediatric COVID-19 vaccine hesitancy and RCIs hesitancy. From November 2021 to July 2022, we observed a 15.8 percentage point increase in the proportion of parents who expressed they were “definitely not getting” or “probably not getting” their children vaccinated against COVID-19. This increase in parental hesitancy for pediatric COVID-19 vaccines may be linked to the rapid development and fast-tracked approval process of pediatric COVID-19 vaccines [23]. Moreover, parents’ socio-behavioral characteristics, including perceptions that pediatric COVID-19 vaccines were unsafe and unimportant, likely contributed to the increased hesitancy for pediatric COVID-19 vaccines. For instance, some parents’ reluctance to vaccinate their children may have been due to rare cases of vaccine-associated adverse events (i.e., myocarditis and pericarditis) following pediatric COVID-19 vaccine administration among adolescents [31]. In addition, some parents believed their children were at a low risk of getting infected, while others reported a lack of family and social support for COVID-19 vaccinations. At the same time, some parents expressed a lack of regret for failing to get their children vaccinated against COVID-19. During the early pandemic, parents’ psychosocial characteristics may have been influenced by vaccine misinformation and misconceptions [32]. On the other hand, studies have shown that beliefs in vaccine safety and effectiveness increased vaccine uptake [33]. Our analysis suggests that parents who perceived vaccines as safe, effective, and important and reported positive family/social influences were more likely to accept pediatric COVID-19 vaccines and RCIs. Our research findings align with other observational studies that indicate parents’ beliefs, risk perceptions, regret, and personal/family experiences regarding vaccines and infectious diseases significantly influence vaccination behavior [30,34].

Between November 2021 and May 2022, following the introduction of pediatric COVID-19 vaccines, there was a temporary increase in the percentage of parents who expressed they were “very hesitant” or “somewhat hesitant” towards RCIs. This trend plateaued and subsequently declined. Although vaccination is widely recognized as a significant achievement in public health, the recent (i.e., 2020–2022) scientific literature supports this transient upward trend in parental reluctance to consent to some routine childhood vaccines [8,35]. The transient rise in parental hesitancy towards RCIs following the introduction of pediatric COVID-19 vaccines may have been caused by the spike in vaccine misinformation and politicized debates about COVID-19 vaccines [23]. False information that claimed that COVID-19 vaccines could adversely impact female fertility and alter human DNA was widely disseminated but later debunked [36]. Parental hesitancy towards pediatric COVID-19 vaccines, conflicting health information and misinformation, cultural and political factors, and parents’ fears/concerns may have influenced this transient rise in parental hesitancy towards RCIs. During this period, no linear trends were evident for RCIs hesitancy for most demographic, behavioral, and social characteristics examined. However, RCIs hesitancy increased among children whose parents had received a recommendation for their child’s COVID-19 vaccination from a healthcare provider and decreased among children who never/rarely/sometimes wore masks. It is uncertain why these correlations occurred. However, multiple factors, such as parents’ adherence to provider recommendations, school policies on mask usage, and children’s compliance with putting on masks, could have influenced these variables. For instance, while some states had policies about mask usage in schools, other states had none in place.

Previous studies have shown that parents’ decision-making regarding pediatric uptake for COVID-19 and other childhood vaccines is influenced by socio-demographic indicators [27,37,38,39]. This scientific evidence is also supported by our study, which found that mothers with a high school education were more likely to be hesitant to vaccinate their children against COVID-19 compared to mothers with a college degree. Parents of children residing in rural areas expressed a higher degree of COVID-19 vaccine hesitancy than parents in urban areas. Furthermore, pediatric COVID-19 vaccine hesitancy was most prevalent among parents of children who were White (43.2%) compared to Black (29.3%) or Hispanic (26.9%). In comparison, RCIs hesitancy was most prevalent among parents of children who were Black (32.0%) compared to Hispanic (24.5%) or White (23.6%). Although RCIs hesitancy was most prevalent among parents of children who were Black, this group was likely more receptive to vaccinating their children against COVID-19 compared to parents of children who were White. This outcome could partly be due to the disproportionately higher COVID-19-related hospitalizations and mortality rates among Black persons during the early stages of the pandemic [40,41]. The African American community has also reported a higher risk perception of COVID-19 infections compared to other racial and ethnic groups [8]. However, historical mistreatment, health disparities [40,42], and the mistrust of healthcare systems may have negatively impacted the uptake of RCIs among African American communities, as shown by their prevalence of RCIs hesitancy, which was higher than the other racial groups in this study.

Additionally, our study found a positive correlation between parental hesitancy towards pediatric COVID-19 vaccines and parental hesitancy towards RCIs across all demographic and psychosocial characteristics examined. This correlation (as depicted by PR estimates) between hesitancy for pediatric COVID-19 vaccines and RCIs tended to be stronger for parent groups with a lower prevalence of COVID-19 vaccine hesitancy among RCIs hesitant and non-hesitant groups, e.g., parents of children who were Asian. On the other hand, the correlation was weaker for groups with a higher prevalence of COVID-19 vaccine hesitancy among RCIs hesitant and non-hesitant groups, e.g., parents of children residing in rural areas. Weaker correlations, a lower PR, and higher COVID-19 vaccine hesitancy may reflect that certain parent groups are more likely to have specific, unique concerns about pediatric COVID-19 vaccines.

This study has several limitations. This cross-sectional study presents a “snapshot” over a 9-month study period of the prevalence of pediatric vaccine hesitancy. Despite this, our findings are consistent with a similar longitudinal study that found a significant increase in the percentage of U.S. parents who believed that childhood vaccines had harmful side effects and may lead to illness or death. Notably, this study was conducted between April 2020 and March 2022, which aligns with our study timeframe (i.e., November 2021–May 2022) [43]. Moreover, our study could not infer cause and effect relationships (i.e., the relative contribution of causality that could indicate that COVID-19 vaccine hesitancy caused RCIs hesitancy). The NIS-CCM relies on parents to report their children’s COVID-19 vaccination status) and does not obtain information from health providers to verify children’s COVID-19 vaccination coverage. As a result, data may be subject to recall bias and social desirability bias. Additionally, there may be some bias in the data estimates after weighting due to the relatively low response rate (20.4%), which is similar to other NIS surveys. However, survey weights are calibrated based on the COVID-19 vaccine administration data reported to the CDC by jurisdictions, which minimizes potential bias from non-response, incomplete sampling frame (i.e., exclusion of households with no phone service), and misclassification of vaccination coverage estimates due to poor/incomplete parents’ recollection. It is important to note that we lacked information on how parents interpreted the RCIs hesitancy question within the context of newly available pediatric COVID-19 vaccines. It is possible that some parents considered the pediatric COVID-19 vaccine as an RCI, which may have contributed to the initial increase in RCIs hesitancy. Also, some parents who were not previously hesitant about RCIs may have reported hesitancy solely due to their concerns about pediatric COVID-19 vaccines. The RCIs hesitancy question was modified in October 2022 to address this issue and expanded into three questions for this age group—hesitancy toward the COVID-19 vaccine, hesitancy toward the influenza vaccine, and hesitancy toward other RCIs. Despite these limitations, this study is of significant public health importance given its assessment of period prevalence, trends, and its depiction of the correlation between parental hesitancy towards COVID-19 vaccines and RCIs using nationally representative data.

Pediatric vaccine hesitancy, along with pandemic disruptions, misinformation, access barriers, politicized debates, and negative perceptions about vaccines, can significantly and adversely impact trusted routine childhood vaccines and new vaccines such as COVID-19 vaccines [23]. Future research is necessary to investigate possible incremental trends in parental hesitancy among different sociodemographic subgroups and examine potentially lower vaccination coverage among children in other age groups, specifically those between 19 and 35 months [44]. Moreover, conducting more in-depth studies to evaluate the impact of introducing new vaccines on parental RCIs hesitancy is essential. As previous pandemics and mass vaccination programs have demonstrated, robust planning and implementation are needed to promote vaccine confidence and ensure public acceptance of vaccines.

## Figures and Tables

**Table 1:** Overall prevalence estimates for pediatric COVID-19 vaccine hesitancy and RCIs hesitancy by demographic indicators and psycho-social characteristics among parents of children aged 5–11 years, United States, November 2021–July 2022, National Immunization Survey—Child COVID Module.

	Sample Distribution	Pediatric COVID-19 Vaccine Hesitancy ^a^	RCIs Hesitancy ^b^
Characteristics	Weighted % (95% CI)	Weighted % (95% CI)	(95% CI)
**Child’s Age (years)**			
5–6 (Referent)	25.3 (24.7–26.0)	36.2 (34.7–37.7)	25.9 (24.5–27.3)
7–9	40.0 (39.2–40.7)	36.0 (34.8–37.2)	24.8 (23.7–25.9)
10–11	34.7 (34.0–35.5)	33.3 (32.0–34.6)	24.4 (23.3–25.7)
**Child’s Race/Ethnicity**			
Hispanic	27.7 (26.9–28.4)	26.9 (25.3–28.5)	24.5 (23.0–26.0)
White, non-Hispanic (Referent)	48.4 (47.6–49.1)	43.2 (42.1–44.2)	23.6 (22.7–24.5)
Black, non-Hispanic	13.9 (13.3–14.4)	29.3 (27.3–31.4)	32.0 (30.0–34.1)
Asian, non-Hispanic	3.7 (3.4–3.9)	11.7 (9.3–14.6)	18.8 (16.2–21.7)
Multiple races/other, non-Hispanic	6.5 (6.1–6.8)	35.5 (32.9–38.2)	25.8 (23.4–28.4)
**Relationship of Respondent to Child**			
Mother (Referent)	56.2 (55.5–57.0)	34.6 (33.6–35.7)	24.2 (23.3–25.2)
Father	32.2 (31.5–32.9)	37.9 (36.6–39.2)	27.3 (26.0–28.5)
Other	11.6 (11.1–12.2)	29.4 (27.2–31.7)	21.6 (19.6–23.8)
**Mother’s Educational Level**			
<High school (Referent)	12.0 (11.4–12.6)	31.3 (28.7–34.0)	26.5 (24.0–29.1)
High school or equivalent	22.1 (21.5–22.8)	40.6 (38.9–42.3)	29.4 (27.8–31.0)
Some college/vocational	31.6 (30.9–32.4)	39.9 (38.5–41.3)	27.4 (26.1–28.6)
≥College degree	34.3 (33.6–34.9)	28.4 (27.3–29.5)	19.4 (18.5–20.4)
**Urban–Rural Residence (Metropolitan Statistical Area (MSA) Status) ^c^**			
Urban (MSA, principal city) (Referent)	32.3 (31.6–33.1)	28.9 (27.6–30.2)	24.4 (23.2–25.6)
Suburban (MSA, non-principal city)	54.3 (53.5–55.1)	35.0 (34.0–36.1)	24.1 (23.2–25.1)
Rural (non-MSA)	13.4 (12.9–13.9)	51.3 (49.2–53.3)	30.1 (28.2–32.1)
**Poverty Status ^d^**			
>Poverty, >$75,000/year (Referent)	37.4 (36.7–38.2)	34.3 (33.1–35.5)	21.4 (20.4–22.4)
>Poverty, ≤$75,000/year	25.0 (24.3–25.7)	37.6 (36.0–39.1)	28.6 (27.1–30.1)
Below poverty level	13.8 (13.2–14.4)	33.1 (30.9–35.4)	27.2 (25.1–29.3)
Income not reported	23.8 (23.1–24.5)	34.9 (33.4–36.5)	25.6 (24.2–27.1)
**Number of Children Under 18 in Household**			
1 child (Referent)	23.5 (22.9–24.1)	31.3 (30.0–32.7)	26.7 (25.4–28.0)
2–3 children	64.4 (63.6–65.1)	34.7 (33.8–35.7)	24.0 (23.2–24.9)
≥4 children	12.1 (11.6–12.7)	44.2 (41.7–46.8)	26.5 (24.3–28.8)
**Social Vulnerability Index (SVI) of County of Residence ^e^**			
Low SVI (Referent)	27.1 (26.5–27.8)	32.9 (31.6–34.3)	21.1 (20.0–22.3)
Moderate SVI	37.2 (36.5–38.0)	35.8 (34.6–37.1)	25.0 (23.8–26.1)
High SVI	35.6 (34.9–36.4)	35.8 (34.4–37.2)	27.2 (25.8–28.5)
**Health and Human Services (HHS) Region**			
Region I: CT, ME, MA, NH, RI, VT (Referent)	3.9 (3.7–4.0)	24.7 (22.7–26.9)	20.9 (19.0–22.9)
Region II: NJ, NY, PR, VI	8.8 (8.4–9.2)	27.1 (24.7–29.7)	24.8 (22.5–27.2)
Region III: DE, DC, MD, PA, VA, WV	8.9 (8.6–9.2)	32.3 (30.5–34.3)	22.9 (21.2–24.6)
Region IV: AL, FL, GA, KY, MS, NC, SC, TN	19.7 (19.1–20.3)	42.3 (40.5–44.1)	28.8 (27.2–30.6)
Region V: IL, IN, MI, MN, OH, WI	15.7 (15.2–16.2)	38.4 (36.4–40.4)	24.7 (23.0–26.5)
Region VI: AR, LA, NM, OK, TX	14.6 (14.1–15.1)	35.5 (33.7–37.4)	25.1 (23.4–26.8)
Region VII: IA, KS, MO, NE	4.5 (4.3–4.7)	42.9 (40.1–45.8)	21.9 (19.6–24.4)
Region VIII: CO, MT, ND, SD, UT, WY	4.0 (3.9–4.2)	37.5 (35.0–40.1)	22.2 (20.0–24.6)
Region IX: AZ, CA, HI, NV, GU	15.6 (14.9–16.2)	27.6 (25.3–30.1)	24.4 (22.2–26.8)
Region X: AK, ID, OR, WA	4.4 (4.1–4.6)	36.4 (33.5–39.4)	24.0 (21.4–26.8)
**Concerned about child getting COVID-19 infection? ^f^**			
Not at all concerned/A little concerned	58.7 (58.0–59.5)	48.0 (47.0–49.0)	26.6 (25.7–27.6)
Moderately concerned/Very concerned (Referent)	41.3 (40.5–42.0)	16.8 (15.8–17.8)	22.6 (21.5–23.7)
**I think pediatric COVID-19 vaccine is safe ^f^**			
Not at all safe/Somewhat safe	56.5 (55.7–57.3)	55.5 (54.3–56.6)	36.4 (35.3–37.5)
Very safe/Completely safe (Referent)	43.5 (42.7–44.3)	5.0 (4.5–5.6)	8.4 (7.7–9.1)
**It is important to get pediatric COVID-19 vaccine to protect my child ^f^**			
Not at all important/A little important	35.9 (35.1–36.6)	78.9 (77.8–80.0)	38.8 (37.5–40.2)
Somewhat important/Very important (Referent)	64.1 (63.4–64.9)	10.2 (9.6–10.9)	16.9 (16.2–17.7)
**Family/friends have gotten pediatric COVID-19 vaccine for their children aged 5–11 years ^f^**			
None/Some	72.0 (71.3–72.7)	46.7 (45.7–47.7)	30.0 (29.1–30.9)
Many/Almost All (Referent)	28.0 (27.3–28.7)	7.0 (6.2–7.9)	12.6 (11.6–13.6)
**My health provider has given recommendation to get pediatric COVID-19 vaccine for my child**			
Yes	34.4 (33.6–35.1)	21.6 (20.5–22.8)	20.5 (19.4–21.6)
No (Referent)	65.7 (64.9–66.4)	42.9 (41.9–43.9)	27.5 (26.6–28.5)
**Child’s school requires pediatric COVID-19 vaccine for in-person classes**			
Yes	4.5 (4.2–4.8)	6.9 (4.9–9.6)	22.2 (19.2–25.6)
No (Referent)	92.9 (92.5–93.3)	36.9 (36.1–37.7)	25.0 (24.3–25.8)
Not in school, Home schooled	2.6 (2.4–2.9)	47.0 (42.0–52.1)	33.6 (28.8–38.8)
**Parental regret if pediatric COVID-19 vaccine is not obtained for my child ^f^**			
Do not agree/Somewhat agree	70.6 (69.9–71.3)	49.2 (48.2–50.2)	31.4 (30.5–32.3)
Strongly agree/Very strongly agree (Referent)	29.4 (28.7–30.1)	3.1 (2.6–3.8)	9.8 (9.0–10.7)
**In the last 7 days, my child has worn a mask when going into indoor public spaces ^f^**			
Never/Rarely/Sometimes	43.8 (43.0–44.5)	51.8 (50.6–52.9)	28.2 (27.2–29.3)
Often/Always (Referent)	56.2 (55.5–57.0)	22.0 (21.0–22.9)	22.4 (21.5–23.4)

Abbreviations: RCIs = Routine Childhood Immunizations; CI = Confidence Interval. The denominator of the percentages represents all completed parent interviews for children aged 5–11-years collated monthly between November 2021–July 2022. ^a^ Pediatric COVID-19 vaccine hesitancy = unvaccinated (and probably or definitely not getting vaccine). Pediatric COVID-19 vaccine hesitancy was assessed with the following question: “Once your child is eligible, how likely are you to get a COVID-19 vaccine?” Participants’ responses were “definitely not getting vaccine”, “probably not getting vaccine”, “probably getting vaccine”, and “definitely getting vaccine”. Responses were dichotomized into two categories: pediatric COVID-19 vaccine hesitancy and pediatric COVID-19 vaccine non-hesitancy. ^b^ RCIs hesitancy = somewhat hesitant or very hesitant to RCIs. RCIs hesitancy was assessed with the following question: “How hesitant about childhood shots would you consider yourself to be?” Response options were “not at all hesitant”, “not that hesitant”, “somewhat hesitant”, and “very hesitant”. Participants’ responses were dichotomized into RCIs hesitancy and RCIs non-hesitancy. ^c^ MSA status was determined based on household reported city and county of residence and was grouped into three categories: MSA principal city = urban; MSA non-principal city = suburban; and non-MSA = rural. MSAs and principal cities were as defined by the U.S. Census Bureau at https://www.census.gov/programs-surveys/metro-micro.html, accessed on 23 April 2024. Non-MSA areas include urban populations not located within an MSA and completely rural areas. ^d^ Income/Poverty level was defined based on total family income in the past calendar year, and the U.S. Census poverty thresholds for that year specified for the applicable family size and the number of children <18 years. Poverty thresholds are available at https://www.census.gov/data/tables/time-series/demo/income-poverty/historical-poverty-thresholds.html, accessed on 23 April 2024. ^e^ The CDC/ATSDR Social Vulnerability Index was developed using 15 U.S. census variables to help officials identify communities needing support before, during, or after disasters. Categorization of NIS-CCM data into an SVI level was based on the zip code of residence reported by the respondent. Details on the SVI are available at https://www.atsdr.cdc.gov/placeandhealth/svi/index.html, accessed on 23 April 2024. ^f^ Response options were dichotomized into two categories. Nonoverlapping 95% confidence intervals (CIs) determined statistically significant estimates between groups.

**Table 2:** Overall estimates and prevalence ratios comparing pediatric COVID-19 vaccine hesitancy within the RCIs hesitant group to pediatric COVID-19 vaccine hesitancy within the RCIs non-hesitant group among parents of children ages 5–11 years (logistic regression analysis), November 2021–July 2022, National Immunization Survey—Child COVID Module.

Characteristics	Pediatric COVID-19 Vaccine Hesitancy			
	RCIs Hesitancy Group ^a^	RCIs Non-Hesitancy Group ^b^	Prevalence Ratio ^c^	
	Weighted % (95% CI)	Weighted % (95% CI)	Unadjusted (95% CI)	Adjusted ^d^ (95% CI)
**Child’s Age (years)**				
5–6	57.0 (53.8–60.1)	28.8 (27.2–30.5)	1.98 (1.83–2.14)	1.88 (1.74–2.04)
7–9	56.4 (53.8–58.9)	29.0 (27.7–30.4)	1.94 (1.82–2.07)	1.89 (1.78–2.02)
10–11	57.1 (54.3–59.9)	25.5 (24.2–26.9)	2.24 (2.08–2.41)	2.17 (2.02–2.33)
**Child’s Race/Ethnicity**				
Hispanic	44.3 (40.7–47.9)	21.2 (19.5–22.9)	2.09 (1.87–2.34)	2.06 (1.84–2.31)
White, non-Hispanic	70.3 (68.3–72.3)	34.5 (33.4–35.7)	2.04 (1.95–2.13)	1.93 (1.84–2.02)
Black, non-Hispanic	46.2 (42.4–50.2)	21.3 (19.1–23.6)	2.18 (1.90–2.49)	2.10 (1.83–2.41)
Asian, non-Hispanic	27.0 (19.6–35.9)	8.2 (6.0–11.2)	3.30 (2.13–5.10)	2.97 (1.99–4.44)
**Relationship of Respondent to Child**				
Mother	56.0 (53.8–58.1)	27.8 (26.7–28.9)	2.02 (1.91–2.13)	1.95 (1.84–2.06)
Father	60.8 (58.1–63.4)	29.1 (27.6–30.6)	2.09 (1.96–2.24)	2.03 (1.90–2.17)
Other	47.2 (41.8–52.7)	24.4 (22.1–27.0)	1.93 (1.66–2.25)	1.96 (1.70–2.27)
**Mother’s Educational Level**				
<High school	50.0 (44.1–55.2)	25.2 (22.4–28.2)	1.97 (1.68–2.32)	1.81 (1.55–2.11)
High school or equivalent	57.6 (54.4–60.8)	33.5 (31.5–35.4)	1.72 (1.59–1.87)	1.70 (1.57–1.83)
Some college/vocational	59.0 (56.2–61.6)	32.5 (30.9–34.1)	1.81 (1.70–1.94)	1.82 (1.70–1.94)
≥College degree	56.5 (53.7–59.2)	21.5 (20.3–22.6)	2.63 (2.45–2.83)	2.59 (2.41–2.78)
**Urban–Rural Residence (Metropolitan Statistical Area (** **MSA) Status) ^e^**				
Urban (MSA, principal city)	47.4 (44.4–50.3)	23.0 (21.6–24.4)	2.06 (1.89–2.25)	2.01 (1.84–2.19)
Suburban (MSA, non-principal city)	59.2 (56.9–61.4)	27.2 (26.1–28.4)	2.17 (2.06–2.30)	2.13 (2.02–2.26)
Rural (Non-MSA)	68.3 (64.6–71.9)	43.5 (41.1–45.9)	1.57 (1.46–1.70)	1.55 (1.43–1.67)
**Poverty Status ^f^**				
>Poverty, ≥$75,000/year	61.1 (58.3–63.7)	26.8 (25.5–28.1)	2.28 (2.14–2.43)	2.18 (2.05–2.33)
>Poverty, <$75,000/year	56.4 (53.3–60.0)	30.0 (28.3–31.8)	1.88 (1.73–2.04)	1.87 (1.73–2.02)
Below poverty level	45.3 (40.8–4.98)	28.7 (26.2–31.4)	1.58 (1.38–1.80)	1.48 (1.30–1.69)
Income not reported	58.5 (55.3–61.7)	26.6 (24.9–28.3)	2.20 (2.02–2.40)	2.12 (1.95–2.31)
**Number of Children Under 18 in Household**				
1 child	50.0 (48.0–53.8)	24.0 (22.7–25.5)	2.12 (1.95–2.30)	2.04 (1.88–2.21)
2–3 children	57.5 (55.4–59.6)	27.4 (26.4–28.4)	2.10 (1.99–2.21)	2.03 (1.93–2.14)
≥4 children	64.5 (59.5–69.2)	37.0 (34.2–40.0)	1.74 (1.56–1.94)	1.70 (1.53–1.89)
**Social Vulnerability Index (SVI) of County of Residence ^g^**				
Low SVI	60.2 (57.1–63.2)	25.5 (24.2–27.0)	2.36 (2.19–2.54)	2.27 (2.11–2.45)
Moderate SVI	57.9 (55.2–60.6)	28.4 (27.0–29.8)	2.04 (1.91–2.18)	1.95 (1.82–2.08)
High SVI	53.7 (50.8–56.6)	29.0 (27.4–30.6)	1.86 (1.72–2.00)	1.80 (1.67–1.95)
**Human Health Services (HHS) Region**				
Region I: CT, ME, MA, NH, RI, VT	51.0 (45.9–56.2)	17.6 (15.6–19.8)	2.90 (2.48–3.40)	2.68 (2.28–3.15)
Region II: NJ, NY, PR, VI	46.4 (41.0–52.0)	20.5 (17.9–23.3)	2.27 (1.90–2.71)	2.16 (1.80–2.58)
Region III: DE, DC, MD, PA, VA, WV	56.5 (52.2–60.7)	25.2 (23.2–27.4)	2.24 (2.00–2.51)	2.16 (1.93–2.42)
Region IV: AL, FL, GA, KY, MS, NC, SC, TN	61.9 (58.3–65.3)	34.3 (32.2–36.4)	1.80 (1.66–1.96)	1.80 (1.66–1.95)
Region V: IL, IN, MI, MN, OH, WI	63.8 (59.7–67.7)	30.1 (27.9–32.3)	2.12 (1.93–2.34)	2.07 (1.88–2.28)
Region VI: AR, LA, NM, OK, TX	53.7 (49.7–57.6)	29.4 (27.4–31.5)	1.83 (1.65–2.02)	1.78 (1.61–1.97)
Region VII: IA, KS, MO, NE	63.4 (57.0–69.3)	36.9 (33.7–40.1)	1.72 (1.51–1.96)	1.67 (1.46–1.91)
Region VIII: CO, MT, ND, SD, UT, WY	69.5 (63.9–74.7)	28.2 (25.6–30.8)	2.47 (2.19–2.79)	2.26 (2.01–2.55)
Region IX: AZ, CA, HI, NV, GU	46.6 (41.2–52.1)	21.3 (18.8–24.0)	2.19 (1.85–2.60)	2.05 (1.73–2.43)
Region X: AK, ID, OR, WA	59.8 (53.3–66.1)	28.6 (25.5–31.9)	2.09 (1.79–2.44)	1.99 (1.71–2.32)
**Concerned about child getting COVID-19 infection? ^h^**				
Not at all concerned/A little concerned	71.8 (69.9–73.5)	39.3 (38.1–40.5)	1.83 (1.76–1.90)	1.78 (1.71–1.85)
Moderately concerned/Very concerned	31.4 (28.9–34.0)	12.3 (11.4–13.3)	2.55 (2.28–2.86)	2.39 (2.13–2.69)
**I think pediatric COVID-19 vaccine is safe ^h^**				
Not at all safe/Somewhat safe	66.8 (64.9–68.7)	48.9 (47.4–50.4)	1.37 (1.31–1.42)	1.35 (1.29–1.40)
Very safe/Completely safe	10.5 (8.0–13.6)	4.5 (3.9–5.0)	2.35 (1.75–3.15)	2.28 (1.70–3.06)
**It is important to get pediatric COVID-19 vaccine to protect my child ^h^**				
Not at all important/A little important	84.9 (83.3–86.5)	75.1 (73.5–76.6)	1.13 (1.10–1.16)	1.13 (1.10–1.16)
Somewhat important/Very important	20.6 (18.6–22.7)	8.1 (7.5–8.7)	2.55 (2.25–2.89)	2.40 (2.11–2.73)
**Family/friends have obtained pediatric COVID-19 vaccine for their children aged 5–11 years ^h^**				
None/Some	63.2 (61.4–65.0)	39.6 (38.4–40.7)	1.60 (1.53–1.66)	1.58 (1.52–1.65)
Many/Almost All	22.1 (18.5–26.2)	4.7 (4.1–5.5)	4.66 (3.71–5.85)	4.49 (3.53–5.71)
**My health provider has given recommendation to get pediatric COVID-19 vaccine for my child**				
Yes	47.2 (44.1–50.4)	14.8 (13.8–16.0)	3.19 (2.88–3.52)	2.95 (2.66–3.27)
No	60.9 (59.0–62.8)	36.0 (34.8–37.2)	1.69 (1.62–1.77)	1.68 (1.60–1.75)
**Child’s school requires pediatric COVID-19 vaccine for in-person classes.**				
Yes	15.8 (9.8–24.5)	4.0 (2.7–7.0)	3.60 (1.87–6.96)	3.28 (1.80–5.99)
No	58.6 (56.9–60.3)	29.5 (28.6–30.4)	1.99 (1.91–2.07)	1.93 (1.85–2.01)
Not in school, Home schooled	73.1 (65.0–79.9)	32.7 (27.3–38.7)	2.23 (1.82–2.74)	2.19 (1.81–2.66)
**Parental regret if pediatric COVID-19 vaccine is not obtained for my child ^h^**				
Do not agree/Somewhat agree	63.1 (61.4–64.8)	42.7 (41.5–43.8)	1.48 (1.42–1.54)	1.48 (1.42–1.53)
Strongly agree/Very strongly agree	13.1 (9.7–17.4)	2.0 (1.6–2.5)	6.56 (4.53–9.50)	5.62 (3.81–8.29)
**In the last 7 days, my child has worn a mask when going into indoor public spaces ^h^**				
Never/Rarely/Sometimes	73.4 (71.4–75.3)	43.0 (41.7–44.3)	1.71 (1.64–1.78)	1.68 (1.61–1.75)
Often/Always	40.4 (38.0–42.8)	16.7 (15.7–17.7)	2.42 (2.23–2.63)	2.29 (2.10–2.49)

Abbreviations: RCIs = Routine Childhood Immunizations; CI = Confidence Interval. Pediatric COVID-19 vaccine hesitancy = unvaccinated [child], definitely not getting vaccine for [child], and probably not getting vaccine for [child]. Pediatric COVID-19 vaccine hesitancy was assessed with, “Once your child is eligible, how likely are you to get a COVID-19 vaccine?” Responses were “definitely not getting vaccine”, “probably not getting vaccine”, “probably getting vaccine”, and “definitely getting vaccine”. Participants’ responses were dichotomized into two categories: pediatric COVID-19 hesitancy and pediatric COVID-19 non-hesitancy. RCI hesitancy = somewhat hesitant and very hesitant. RCI hesitancy was assessed with, “How hesitant about childhood shots would you consider yourself to be?” Responses were “not at all hesitant”, “not that hesitant”, “somewhat hesitant”, and “very hesitant”. Participants’ responses were dichotomized into two categories: RCIs hesitancy and RCIs non-hesitancy. ^a^ RCIs hesitancy group = prevalence estimates of COVID-19 vaccine hesitancy among parents with RCIs hesitancy. ^b^ RCIs non-hesitancy group (referent group) = prevalence estimates of COVID-19 vaccine hesitancy among parents without RCIs hesitancy. ^c^ Prevalence ratio = prevalence of COVID-19 vaccine hesitancy among parents with RCIs hesitancy divided by prevalence of COVID-19 vaccine hesitancy among parents without RCIs hesitancy. ^d^ Adjusted for child age group, child race, relationship of respondent to child, mother’s educational level, MSA status, and poverty status. ^e^ MSA status was determined based on household reported city and county of residence and was grouped into three categories: MSA principal city = urban; MSA non-principal city = suburban; and non-MSA = rural. MSAs and principal cities were as defined by the U.S. Census Bureau at https://www.census.gov/programs-surveys/metro-micro.html, accessed on 23 April 2024. (Non-MSA areas include urban populations not located within an MSA and completely rural areas. ^f^ Income/Poverty level was defined based on total family income in the past calendar year, and the U.S. Census poverty thresholds for that year specified for the applicable family size and number of children <18 years. Poverty thresholds are available at https://www.census.gov/data/tables/time-series/demo/income-poverty/historical-poverty-thresholds.html, accessed on 23 April 2024. ^g^ The CDC/ATSDR Social Vulnerability Index was developed using 15 U.S. census variables to help officials identify communities needing support before, during, or after disasters. Categorization of NIS-CCM data into an SVI level was based on the zip code of residence reported by the respondent. Details on the SVI are available at https://www.atsdr.cdc.gov/placeandhealth/svi/index.html, accessed on 23 April 2024. ^h^ Response options were dichotomized into two categories. Outputs are statistically significant at *p* < 0.05 compared to the referent group. There was consistent statistical association between COVID-19 vaccine and RCIs hesitancy across all demographic and psychosocial factors for both unadjusted and adjusted prevalence ratios.

**Table 3:** Trends in parental hesitancy towards routine childhood immunizations by sociodemographic characteristics and behavioral and social drivers of COVID-19 vaccination as reported by parents of children ages 5–11 years (weighted linear regression analysis), November 2021–July 2022, National Immunization Survey—Child COVID Module.

Characteristics	Average Monthly Percentage Point Change in RCIs Hesitancy (95% CI)	*p* Value
**Total**	0.1 (−0.4–0.7)	0.60
**Child’s Age (years)**		
5–6	−0.3 (−1.1–0.6)	0.51
7–9	0.4 (−0.2–1.0)	0.19
10–11	0.1 (−0.3–0.5)	0.55
**Child’s Race/Ethnicity**		
Hispanic	0.1 (−0.3–0.5)	0.75
White, non-Hispanic	−0.1 (−0.9–0.8)	0.87
Black, non-Hispanic	0.7 (−0.1–1.4)	0.08
Asian, non-Hispanic	1.1 (−0.3–2.5)	0.10
Other/Multiple	−0.1 (−1.1–0.9)	0.78
**Relationship of Respondent to Child**		
Mother	0.3 (−0.2–0.8)	0.22
Father	−0.2 (−0.8–0.5)	0.55
Other	0.0 (−1.1–1.2)	0.95
**Mother’s Educational Level**		
<High school	0.7 (−0.3–1.6)	0.13
High school or equivalent	0.3 (−0.6–1.2)	0.43
Some college/vocational	−0.3 (−0.9–0.4)	0.41
≥College degree	0.2 (−0.4–0.7)	0.54
**Urban–Rural Residence (Metropolitan Statistical Area (** **MSA) Status) ^a^**		
Urban (MSA, principal city)	0.2 (−0.2–0.6)	0.36
Suburban (MSA, non-principal city)	0.1 (−0.5–0.7)	0.72
Rural (non-MSA)	0.2 (−0.9–1.2)	0.71
**Poverty Status ^b^**		
>Poverty, ≥$75,000/year	0.1 (−0.5–0.6)	0.81
>Poverty, <$75,000/year	−0.2 (−1.1–0.6)	0.55
Below poverty level	0.5 (−0.5–1.4)	0.28
Income not reported	0.4 (−0.2–1.0)	0.18
**Number of Children Under 18 in Household**		
1 child	−0.1 (−0.8–0.6)	0.71
2–3 children	0.1 (−0.5–0.7)	0.62
≥4 children	0.6 (−0.3–1.6)	0.14
**Social Vulnerability Index (SVI) of County of Residence ^c^**		
Low SVI	−0.1 (−0.9–0.7)	0.80
Moderate SVI	−0.2 (−1.2–0.8)	0.64
High SVI	0.3 (−0.4–0.9)	0.36
**Human Health Services (HHS) Region**		
Region I: CT, ME, MA, NH, RI, VT	−2.0 (−0.8–0.4)	0.42
Region II: NJ, NY, PR, VI	−0.1 (−0.8–0.5)	0.66
Region III: DE, DC, MD, PA, VA, WV	−0.2 (−1.1–0.6)	0.55
Region IV: AL, FL, GA, KY, MS, NC, SC, TN	0.0 (−0.9–0.8)	0.91
Region V: IL, IN, MI, MN, OH, WI	0.4 (0.0–0.9)	0.06
Region VI: AR, LA, NM, OK, TX	−0.1 (−1.0–0.8)	0.78
Region VII: IA, KS, MO, NE	0.4 (−0.9–1.6)	0.53
Region VIII: CO, MT, ND, SD, UT, WY	0.4 (−0.5–1.4)	0.29
Region IX: AZ, CA, HI, NV, GU	0.5 (−0.6–1.7)	0.28
Region X: AK, ID, OR, WA	0.2 (−1.0–1.3)	0.74
**Concerned about child getting COVID-19 infection? ^d^**		
Not at all concerned/A little concerned	−1.0 (−0.9–0.6)	0.67
Moderately concerned/Very concerned	0.2 (−0.4–0.8)	0.52
**I think pediatric COVID-19 vaccine is safe ^d^**		
Not at all safe/Somewhat safe	−0.2 (−1.1–0.8)	0.66
Very safe/Completely safe	0.3 (−0.1–0.6)	0.16
**It is important to get pediatric COVID-19 vaccine to protect my child ^d^**		
Not at all important/A little important	−0.8 (−2.1–0.5)	0.18
Somewhat important/Very important	0.0 (−0.4–0.4)	0.86
**Family/friends have obtained pediatric COVID-19 vaccine for their children aged 5–11 years ^d^**		
None/Some	0.2 (−0.7–1.2)	0.58
Many/Almost All	0.5 (0.0–0.9)	0.04 *
**My health provider has given recommendation to get pediatric COVID-19 vaccine for my child**		
Yes	0.6 (0.1–1.1)	0.02 *
No	0.0 (−0.9–0.9)	0.96
**Child’s school requires pediatric COVID-19 vaccine for in-person classes.**		
Yes	−0.2 (−1.8–1.3)	0.73
No	0.1 (−0.5–0.6)	0.72
Not in school, Home schooled	2.1 (−0.9–5.2)	0.14
**Parental regret if pediatric COVID-19 vaccine is not obtained for my child ^d^**		
Do not agree/Somewhat agree	−0.1 (−0.9–0.7)	0.70
Strongly agree/Very strongly agree	0.1 (−0.3–0.6)	0.44
**In the last 7 days, my child has worn a mask when going into indoor public spaces ^d^**		
Never/Rarely/Sometimes	−1.2 (−1.8–0.5)	0.00 *
Often/Always	0.3 (0.0–0.7)	0.05 *

Abbreviations: CI = Confidence Interval. Pediatric COVID-19 vaccine hesitancy = unvaccinated [child], definitely not getting vaccine for [child], and probably not getting vaccine for [child]. Pediatric COVID-19 vaccine hesitancy was assessed with, “Once your child is eligible, how likely are you to get a COVID-19 vaccine?” Responses were “definitely not getting vaccine”, “probably not getting vaccine”, “probably getting vaccine”, and “definitely getting vaccine”. Participants’ responses were dichotomized into two categories: pediatric COVID-19 hesitancy and pediatric COVID-19 non-hesitancy. RCIs hesitancy = somewhat hesitant and very hesitant. RCIs hesitancy was assessed with, “How hesitant about childhood shots would you consider yourself to be?” Responses were “not at all hesitant”, “not that hesitant”, “somewhat hesitant”, and “very hesitant”. Participants’ responses were dichotomized into two categories: RCIs hesitancy and RCIs non-hesitancy. ^a^ MSA status was determined based on household reported city and county of residence and was grouped into three categories: MSA principal city = urban; MSA non-principal city = suburban; and non-MSA = rural. MSAs and principal cities were as defined by the U.S. Census Bureau at https://www.census.gov/programs-surveys/metro-micro.html, accessed on 23 April 2024. Non-MSA areas include urban populations not located within an MSA and completely rural areas. ^b^ Income/Poverty level was defined based on total family income in the past calendar year, and the U.S. Census poverty thresholds for that year specified for the applicable family size and number of children <18 years. Poverty thresholds are available at https://www.census.gov/data/tables/time-series/demo/income-poverty/historical-poverty-thresholds.html, accessed on 23 April 2024. ^c^ The CDC/ATSDR Social Vulnerability Index was developed using 15 U.S. census variables to help officials identify communities needing support before, during, or after disasters. Categorization of NIS-CCM data into an SVI level was based on the zip code of residence reported by the respondent. Details on the SVI are available at https://www.atsdr.cdc.gov/placeandhealth/svi/index.html, accessed on 23 April 2024. ^d^ Response options were dichotomized into two categories. * Statistically significant at *p* < 0.05 compared to the referent group.

## Data Availability

The NIS-CCM data can be accessed at the NCHS Research Data Center following an approval process.
